# Expression of Glycolysis-Related Proteins in Cancer of Unknown Primary Origin

**DOI:** 10.3389/fonc.2021.682665

**Published:** 2021-06-24

**Authors:** Murilo Bonatelli, Isabella Fernandes Fornari, Priscila Neves Bernécule, Lara Esquiapatti Pinheiro, Ricardo Filipe Alves Costa, Adhemar Longatto-Filho, João Neif Antonio Junior, Eduardo Caetano Albino Silva, Flávio Mavignier Cárcano, Céline Pinheiro

**Affiliations:** ^1^ Molecular Oncology Research Center, Barretos Cancer Hospital, Barretos, Brazil; ^2^ Barretos School of Health Sciences Dr. Paulo Prata—FACISB, Barretos, Brazil; ^3^ Research and Teaching Institute, Barretos Cancer Hospital, Barretos, Brazil; ^4^ Life and Health Sciences Research Institute (ICVS), School of Medicine, University of Minho, Braga, Portugal; ^5^ ICVS/3B’s-PT Government Associate Laboratory, Braga, Portugal; ^6^ Laboratory of Medical Investigation (LIM-14), School of Medicine, University of São Paulo, São Paulo, Brazil; ^7^ Medical Oncology Department, Barretos Cancer Hospital, Barretos, Brazil; ^8^ Pathology Department, Barretos Cancer Hospital, Barretos, Brazil

**Keywords:** immunohistochemistry, glycolytic metabolism, metabolic reprogramming, Warburg effect, cancer of unknown primary origin

## Abstract

**Introduction:**

Cancer of unknown primary origin (CUP) is defined as metastatic cancer without identification of the primary site. Considering that only 15–20% of patients with CUP show a favorable outcome, identifying biomarkers may help improve the clinical management of patients who do not respond well to conventional therapies. In this context, the study of the metabolic profile of CUP may pave the way to establish new biomarkers and/or therapeutic targets; therefore, this study aimed to characterize the expression of metabolism-related proteins in CUP.

**Materials and Methods:**

The expression of monocarboxylate transporters MCT1, MCT2 and MCT4, their chaperone CD147, the glucose transporter GLUT1 and the pH regulator CAIX was evaluated by immunohistochemistry in a series of 118 CUP patients, and the results were associated with the available clinicopathological information.

**Results:**

The metabolism-related proteins MCT1, MCT4, CD147, GLUT1 and CAIX were expressed in a critical portion of the CUP (approximately 20 to 70%). MCT1 and CD147 were both more frequently expressed in cases with lymph nodes as metastasis dominant sites (*p* = 0.001) as well as in samples from lymph nodes (*p <*0.001 and *p* = 0.002, respectively), while MCT1 expression was more frequently expressed in squamous cell carcinomas (*p* = 0.045). A higher overall survival was observed in patients with tumors positive for GLUT1 and CAIX expression (*p* = 0.011 and *p* = 0.041, respectively), but none of the proteins was an independent prognostic factor for overall survival in multivariable analysis.

**Conclusion:**

The results suggest that a portion of CUPs present a hyperglycolytic phenotype, which is associated with higher overall survival.

## Introduction

Cancer of unknown primary origin (CUP) is defined as metastatic cancer without identification of the primary site, even after careful evaluation (1). In the early 1990s, CUP accounted for 3–5% of all cancer diagnoses; however, advances in radiological and molecular aspects have improved the identification of primary tumor sites, decreasing the diagnostic rate of CUP to 1–2% (2, 3). The investigation of this tumor type includes a detailed physical examination, laboratory tests, histopathological review of the material used for immunohistochemistry, and pelvic, thorax and abdominal computed tomography (1). CUP has a very heterogeneous clinical presentation, unpredictable metastatic spread, high aggressiveness and a low response to chemotherapy, presenting very low survival, with only 15–20% of patients showing a favorable outcome (4). For patients involved in clinical trials, the median survival is 6–10 months, and fewer than 25% of patients survive more than one year. For those who are not enrolled in clinical trials, the survival rates decrease to 2–3 months (5). Importantly, despite advances in the diagnosis and therapy fields for CUP patients, the mechanisms of carcinogenesis, development and progression of these tumors remain unclear (3, 6).

Metabolic rewiring in tumors is gaining increased attention and is a hallmark of cancer (7). This phenomenon has been described as the Warburg effect or aerobic glycolysis (8, 9), in which tumor cells preferentially produce energy through glycolysis rather than mitochondrial oxidative phosphorylation (10). Although aerobic glycolysis is far less efficient than oxidative phosphorylation for ATP production, this metabolic adaptation is required to use other carbon source pathways as building blocks for biomolecule syntheses, which are essential for cell proliferation. The accumulation of these important portions of carbons as glycolytic intermediates feeds anabolic pathways, producing proteins, lipids and nucleic acids and maintaining an increased state of cellular proliferation, which favors tumor cell survival over normal cells (11, 12). Furthermore, the loss of a combination of tumor suppressor genes, such as *TP53*, and activation of oncogenes (oncogene addiction), particularly related to the PI3K-AKT axis, which is linked to both growth control and glucose metabolism, are intrinsic tumor mechanisms in the orchestration of metabolic reprogramming (11). Several proteins play an important role in the metabolic reprogramming of tumor cells (13), such as glucose transporter 1 (GLUT1), a transmembrane protein that facilitates glucose influx (14, 15), monocarboxylate transporters (MCTs), particularly isoforms 1 and 4, because of their function in lactate efflux (16) in close association with their chaperone CD147 (17), and carbonic anhydrase 9 (CAIX), a pH regulator contributing to preservation of intracellular pH (18). Therefore, these proteins have increased expression and are potential therapeutic targets for cancer treatment (13, 16, 19).

A recent study in CUP has shown differences in the expression profile of proteins related to metabolism, according to the histological subtype and clinical characteristics (20); however, additional studies are warranted to provide robust information on the metabolic characteristics of CUP. The identification of prognostic markers may contribute to the treatment of CUP patients who do not respond well to conventional therapies, providing alternatives to the treatment employed. In this context, the study of the metabolic profile of tumor cells may pave the way to establish new markers and/or therapeutic targets (21). Thus, this study aimed to characterize the expression of MCT1, MCT2, MCT4, CD147, GLUT1 and CAIX in the CUP.

## Materials and Methods

### Case Selection and Clinicopathological Information

The study series included 118 CUP patients diagnosed from 2002 to 2017 at the Barretos Cancer Hospital. The inclusion criteria used were clinical/radiological CUP diagnoses and an immunohistochemical panel according to the discretion of the pathologist (more frequently evaluated markers: cytokeratin 7, cytokeratin 20, thyroid transcription factor-1, vimentin and carcinoembryonic antigen), PSA (prostate-specific antigen) evaluation in men, HCG (human chorionic gonadotropin) evaluation in poorly differentiated carcinomas cases and formalin-fixed paraffin-embedded tissues available at the Pathology Department. Patients were excluded by the following criteria: poorly differentiated squamous cell carcinoma limited to cervical lymph nodes, women with adenocarcinoma limited to axillary lymph nodes, women with adenocarcinoma and involvement of the peritoneal cavity only, young men (<55 years) with middle line tumor growth, neuroendocrine carcinoma, melanoma metastasis, the presence of other primary malignancies (except non-melanoma tumors), positive HIV serology and patients on chronic use of immunosuppressive agents.

Sociodemographic and clinicopathological features included, among others, the age at diagnosis, sex, lifestyle habits (smoking and alcoholism status), tumor histological subtype, first-line treatment and follow-up. The detailed data are presented in **Table 1**. The study data were collected and managed using REDCap electronic data capture tools hosted at the Barretos Cancer Hospital (23, 24). The Barretos Cancer Hospital Ethics Committee approved the present study (1055/2015, CAAE 51579715.0.0000.5437).

**Table 1:** Sociodemographic and clinicopathological characteristics of patients with cancer of unknown primary origin.

Sociodemographic and clinicopathological characteristics	n (%)
**All patients**	**118 (100.0)**
**Age (Mean ± SD = 59.5 ± 12.7 Min–Max = 19.9–88.8)**	
<59.5 years	57 (48.3)
≥59.5 years	61 (51.7)
**Sex**	
Female	63 (53.4)
Male	55 (46.6)
**Smoker**	
Yes	50 (42.4)
No	62 (52.5)
Unknown	6 (5.0)
**Alcoholism**	
Yes	25 (21.2)
No	81 (68.6)
Unknown	12 (10.1)
**Cancer family history**	
Yes	44 (37.2)
No	59 (50.0)
Unknown	15 (12.7)
**Metastasis dominant site**	
Liver	45 (52.4)
Bone	30 (38.1)
Lymph node	27 (22.9)
Lung	3 (2.5)
Central nervous system	2 (1.7)
Others	11 (9.3)
**Biopsy site**	
Liver	40 (33.9)
Bone	27 (22.9)
Lymph node	31 (26.2)
Central nervous system	2 (1.7)
Others	15 (12.7)
Unknown	3 (2.5)
**Histological subtype**	
Adenocarcinoma	67 (56.8)
Carcinoma	13 (11.0)
Squamous cell carcinoma	9 (7.6)
Well-differentiated adenocarcinoma	1 (0.8)
Poorly differentiated adenocarcinoma	8 (6.8)
Poorly differentiated carcinoma	16 (13.5)
Not classified	4 (3.4)
**PS (ECOG)**	
0–1	48 (40.7)
≥2	35 (29.6)
Unknown	35 (29.6)
**First-Line Chemotherapy**	
Etoposide/Cisplatin	1 (0.8)
Carboplatin/Paclitaxel	20 (16.9)
Gemcitabine/Cisplatin	5 (4.2)
Others	25 (21.2)
No Chemotherapy	67 (56.7)

SD, standard deviation; PS (ECOG), performance status (Eastern Cooperative Oncology Group) (22).

### Immunohistochemistry

MCT1, MCT2, MCT4, CD147, GLUT1 and CAIX expression was evaluated by immunohistochemistry in histological sections of cancer of unknown primary origin samples. Detailed information on antigen retrieval and each antibody used are described in **Table 2**. Immunohistochemistry for MCT1 and CD147 was performed using a polymer system (Ultra Vision ONE Detection System: HRP Polymer, Lab Vision Corporation, Fremont, CA) as previously described (25). MCT4 reactions were performed using a streptavidin–biotin–peroxidase complex (Ultravision Detection System: Large Volume Anti-Polyvalent, HRP, Lab Vision Corporation, Fremont, CA), as previously described (26); finally, MCT2, GLUT1 and CAIX reactions were performed using a biotin-free principle (ADVANCE HRP; Dako, Carpinteria, CA) according to the manufacturer’s instructions and as previously described (25). For visualization, the slides were incubated with 3,3’-diaminobenzidine (Liquid DAB+ Substrate Chromogen System; Dako, Carpinteria, CA) according to the manufacturer’s instructions, counterstained with hematoxylin and permanently mounted. As positive controls, squamous cell carcinoma of the oral cavity was used for MCT1 and MCT4, normal kidney for MCT2, normal colon for CD147, placenta for GLUT1 and normal gastric mucosa for CAIX. Negative controls were available in the same tissue sections used as positive controls.

**Table 2:** Detailed aspects of immunohistochemistry.

Protein	Antigen retrieval	Antibody	Clonality	Dilution, incubation time and temperature
**MCT1**	EDTA (1 mM, pH = 8.0), 98°C, 20	AB3538P	Polyclonal	1:300, overnight, RT
		Chemicon International		
**MCT2**	Citrate (0.01 M, pH = 6.0), 98°C, 20 min	sc-50322	Polyclonal	1:800, 2 h, RT
		Santa Cruz Biotechnology		
**MCT4**	Citrate (0.01 M, pH = 6.0), 98°C, 20 min	sc-50329	Polyclonal	1:500, 2 h, RT
		Santa Cruz Biotechnology		
**CD147**	EDTA (1 mM, pH = 8.0), 98°C, 20 min	sc-71038	Monoclonal (1.BB.218)	1:500, overnight, RT
		Santa Cruz Biotechnology		
**GLUT1**	Citrate (0.01 M, pH = 6.0), 98°C, 20 min	ab15309	Polyclonal	1:500, 2 h, RT
		Abcam		
**CAIX**	Citrate (0.01 M, pH = 6.0), 98°C, 20 min	ab15086	Polyclonal	1:4,000, 2 h, RT
		Abcam		

RT, room temperature.

### Immunohistochemical Evaluation

The reactions were scored semi-quantitatively for the extension of expression in cancer cells as follows: 0: no immunoreactive cells; 1: <5% immunoreactive cells; 2: 5–50% immunoreactive cells; and 3: >50% immunoreactive cells. Additionally, the intensity of staining was scored semi-qualitatively as follows: 0: negative; 1: weak; 2: intermediate; and 3: strong. The final score was defined as the sum of both parameters (extension and intensity) and grouped as negative (scores 0–2) and positive (score 3–6), as previously described (27). Only protein expression in the plasma membrane was considered for further analysis. The reactions were evaluated by two pathologists (ECAS and AL).

### Statistical Analysis

The data collected were analyzed using the Statistical Package for Social Sciences (SPSS) software for Windows (version 21.0). Statistical significance for comparisons between biomarker immunoexpression and clinicopathological parameters, as well as co-expression analysis, were evaluated using Pearson’s chi-squared (χ^2^) and Fisher’s exact tests, according to the series characteristics. Kaplan–Meier analysis was used to assess overall survival, and comparisons between survival curves were performed using log-rank tests. The time to event of interest considered was the date of diagnosis until the date of death. Multivariable analysis of survival was performed using the Cox proportional hazards regression model. Independent variables were analyzed by univariable analysis, followed by multivariable analysis of all variables that reached a p-value <0.100 at univariable analysis. The threshold of significant *p* values was established as *p <*0.050.

## Results

### Expression of MCT1, MCT2, MCT4, CD147, GLUT1 and CAIX in Cancer of Unknown Primary Origin Patients

**Figure 1:**
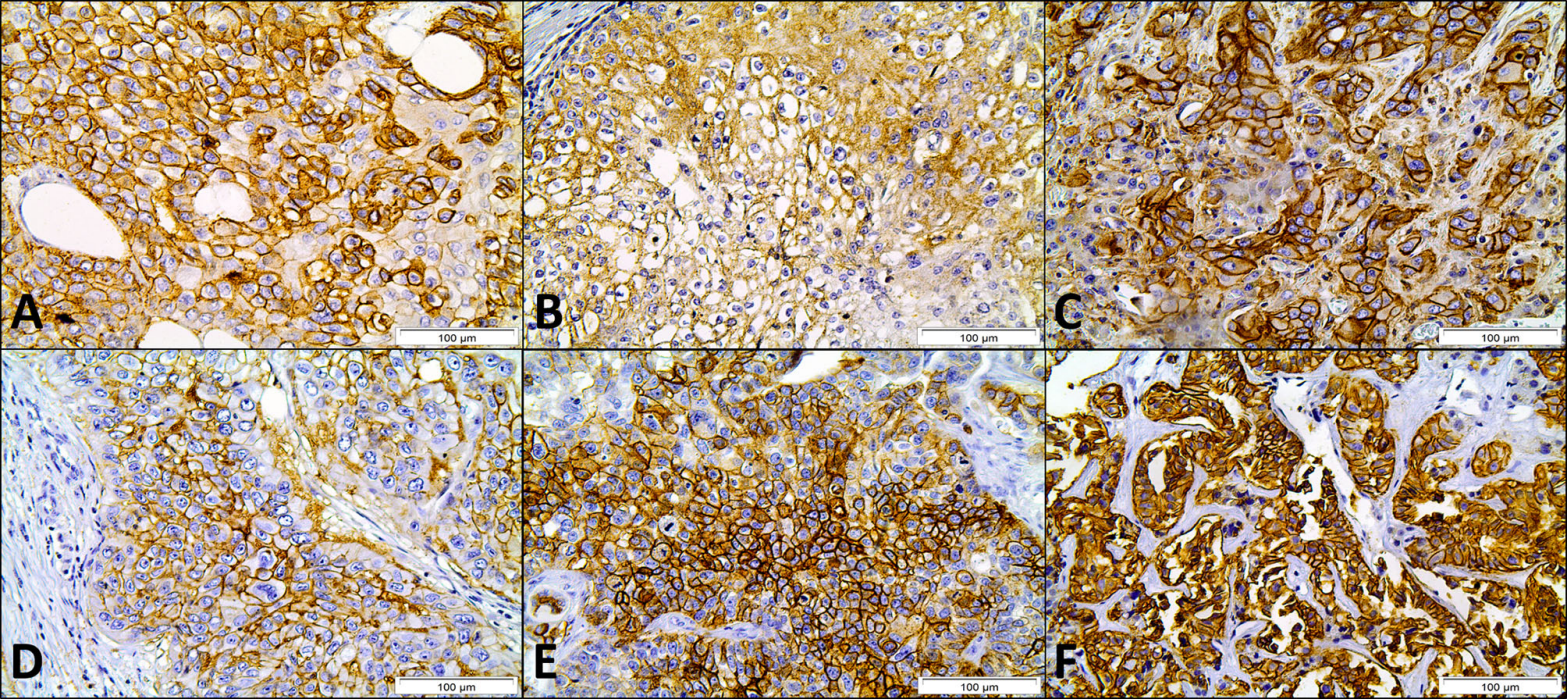
Immunohistochemical expression of MCT1 **(A)**, MCT2 **(B)**, MCT4 **(C)**, CD147 **(D)**, GLUT1 **(E)** and CAIX **(F)** in cancer of unknown primary origin samples. Magnification: 400×.

Immunohistochemical expression of metabolism-related proteins in CUP patients (**Figure 1**) showed that the expression of MCT4, CD147, GLUT1 and CAIX was mostly found in the plasma membrane (approximately 80% of positive samples). For MCT1 and MCT2, plasma membrane expression was found in 52.3 and 33.3% of positive cases, respectively. Considering only plasma membrane expression, MCT1 was found in 23 (19.5%), MCT2 in three (2.5%), MCT4 in 79 (66.9%), CD147 in 40 (33.9%), GLUT1 in 53 (44.9%) and CAIX in 40 (33.9%) CUP samples.

### Clinicopathological Significance of MCTs, CD147, GLUT1 and CAIX in Cancer of Unknown Primary Origin Patients

The associations between the clinicopathological data and expression of metabolism-related proteins in CUP patients are shown in **Table 3**. MCT1 expression was more frequently expressed in cases with lymph nodes as the metastasis dominant site (*p* = 0.001), as well as in biopsies from lymph nodes (*p <*0.001) and in squamous cell carcinomas (*p* = 0.045). The MCT chaperone CD147 was significantly associated with the metastasis dominant site (*p* = 0.001) and biopsy site (*p* = 0.002), following the same frequency pattern of MCT1. MCT2 (data not shown because of a low number of positive cases), MCT4, CD147, GLUT1 and CAIX showed no significant associations with the clinicopathological data.

**Table 3:** Association of MCT1, MCT4, CD147, GLUT1 and CAIX membrane expression with the clinicopathological characteristics of cancer of unknown primary origin patients.

	MCT1			MCT4			CD147			GLUT1			CAIX		
	n	Positive (%)	*p*	n	Positive (%)	*p*	n	Positive (%)	*p*	n	Positive (%)	*p*	n	Positive (%)	*p*
**Age (years)**			0.605			0.309			0.406			0.243			0.782
<59.7	**54**	12 (22.2)		**56**	41 (73.2)		**53**	17 (32.1)		**52**	22 (42.3)		**50**	18 (36.0)	
≥59.7	**60**	11 (18.3)		**59**	38 (64.4)		**58**	23 (39.7)		**58**	31 (53.4)		**57**	22 (38.6)	
**Sex**			0.812			0.716			0.617			0.718			0.083
Female	**62**	12 (19.4)		**61**	41 (67.2)		**59**	20 (33.9)		**58**	27 (46.6)		**58**	26 (44.8)	
Male	**52**	11 (21.2)		**54**	38 (70.4)		**52**	20 (38.5)		**52**	26 (50.0)		**49**	14 (28.6)	
**Smoker**			0.557			0.138			0.379			0.826			0.858
No	**60**	11 (18.3)		**61**	39 (63.9)		**57**	19 (33.3)		**55**	27 (49.1)		**52**	20 (38.5)	
Yes	**48**	11 (22.9)		**48**	37 (77.1)		**48**	20 (41.7)		**49**	23 (46.9)		**49**	18 (36.7)	
**Alcoholism**			0.459			0.910			0.933			0.908			0.116
No	**79**	15 (19.0)		**79**	55 (69.6)		**76**	29 (38.2)		**74**	36 (48.6)		**73**	29 (39.7)	
Yes	**23**	6 (26.1)		**24**	17 (70.8)		**23**	9 (39.1)		**24**	12 (50.0)		**23**	5 (21.7)	
**Cancer family history**			0.957			0.813			0.790			0.192			0.735
No	**58**	13 (22.4)		**57**	41 (71.9)		**55**	20 (36.4)		**54**	23 (42.6)		**52**	19 (36.5)	
Yes	**41**	9 (22.0)		**43**	30 (69.8)		**41**	16 (39.0)		**41**	23 (56.1)		**40**	16 (40.0)	
**Metastasis dominant site**			**0.001**			0.354			**0.001**			0.240			0.501
Liver	**42**	5 (11.9)		**42**	26 (61.9)		**41**	9 (22.0)		**41**	20 (48.8)		**41**	15 (36.6)	
Bone	**30**	2 (6.7)		**30**	19 (63.3)		**28**	7 (25.0)		**27**	9 (33.3)		**26**	7 (26.9)	
Lymph node	**27**	11 (40.7)		**27**	21 (77.8)		**27**	17 (63.0)		**27**	15 (55.6)		**26**	11 (42.3)	
**Biopsy site**			**<0.001**			0.527			**0.002**			0.311			0.660
Liver	**38**	3 (7.9)		**37**	23 (62.2)		**36**	6 (16.7)		**37**	19 (51.4)		**35**	13 (37.1)	
Bone	**27**	2 (7.4)		**27**	17 (63.0)		**25**	8 (32.0)		**24**	8 (33.3)		**25**	7 (28.0)	
Lymph node	**30**	14 (46.7)		**31**	23 (74.2)		**31**	18 (58.1)		**29**	15 (51.7)		**28**	11 (39.3)	
**Histological subtype**			**0.045**			0.972			0.084			0.748			0.738
Adenocarcinoma	**65**	9 (13.8)		**65**	45 (69.2)		**62**	17 (27.4)		**61**	27 (44.3)		**60**	25 (41.7)	
Carcinoma	**13**	5 (38.5)		**12**	8 (66.7)		**12**	5 (41.7)		**13**	7 (53.8)		**12**	3 (25.0)	
Squamous cell carcinoma	**8**	4 (50.0)		**9**	7 (77.8)		**9**	6 (66.7)		**9**	6 (66.7)		**9**	3 (33.3)	
Poorly differentiated adenocarcinoma	**8**	1 (12.5)		**8**	5 (62.5)		**8**	2 (25.0)		**8**	4 (50.0)		**7**	2 (28.6)	
Poorly differentiated carcinoma	**15**	2 (13.3)		**16**	11 (68.8)		**15**	8 (53.8)		**14**	6 (42.9)		**14**	4 (28.6)	
**PS (ECOG)**			0.248*			0.426			0.578			0.550			0.141
0–1	**46**	11 (23.9)		**47**	33 (70.2)		**44**	16 (36.4)		**45**	22 (48.9)		**43**	18 (41.9)	
≥2	**34**	4 (11.8)		**34**	21 (61.8)		**33**	10 (30.3)		**31**	13 (41.9)		**31**	10 (32.3)	

PS (ECOG), performance status (Eastern Cooperative Oncology Group) (22). *Fisher’s exact test. Significant p-values are depicted in bold.

### Co-Expression Analysis

When evaluating the co-expression of MCTs with the other metabolism-related proteins in CUP patients (**Table 4**), we found that MCT1 and MCT4 were significantly co-expressed with CD147 (*p* = 0.001 and *p <*0.001, respectively). Additionally, MCT4 showed significant co-expression with both GLUT1 (*p* = 0.006) and CAIX (*p* = 0.036).

**Table 4:** Co-expression of MCTs with CD147, GLUT1 and CAIX in cancer of unknown primary origin patients.

	MCT1			MCT4		
	n	Positive (%)	*p*	n	Positive (%)	*p*
**CD147**			**0.001**			**<0.001**
Negative	**70**	8 (11.4)		**71**	41 (57.7)	
Positive	**38**	15 (39.5)		**40**	36 (90.0)	
**GLUT1**			0.421			**0.006**
Negative	**55**	10 (18.2)		**56**	33 (58.9)	
Positive	**53**	13 (24.5)		**53**	44 (83.0)	
**CAIX**			0.980			**0.036**
Negative	**65**	14 (21.5)		**67**	42 (62.7)	
Positive	**40**	9 (22.5)		**39**	32 (82.1)	

Significant p-values are depicted in bold.

Regarding the association of the glycolytic phenotype and clinicopathological parameters of CUP patients (**Table 5**), we found that MCT1 and CD147, as well as MCT4 and CD147 co-expression, were more frequently found in cases with lymph nodes as the metastasis dominant site (*p <*0.001) and biopsies from lymph nodes (*p <*0.001).

**Table 5:** Co-expression association of metabolism-related proteins with the clinicopathological characteristics of cancer of unknown primary origin patients.

	MCT1/CD147			MCT4/CD147			MCT4/GLUT1			MCT4/CAIX		
	n	Positive (%)	*p*	n	Positive (%)	*p*	n	Positive (%)	*p*	n	Positive (%)	*p*
**Age (years)**			0.923			0.627			0.273			0.957
<59.7	**55**	7 (12.7)		**54**	16 (29.6)		**54**	18 (33.3)		**52**	15 (28.8)	
≥59.7	**60**	8 (13.3)		**59**	20 (33.9)		**60**	26 (43.3)		**58**	17 (29.3)	
**Sex**			0.663			0.324			0.860			0.439
Female	**52**	6 (11.5)		**52**	19 (36.5)		**53**	20 (37.7)		**51**	13 (25.5)	
Male	**63**	9 (14.3)		**61**	17 (27.9)		**61**	24 (39.3)		**59**	19 (32.2)	
**Smoker**			0.735			0.172			0.441			0.247
No	**61**	9 (14.8)		**59**	16 (27.1)		**59**	21 (35.6)		**56**	14 (25)	
Yes	**48**	6 (12.5)		**48**	19 (39.6)		**49**	21 (42.9)		**48**	17 (35.4)	
**Alcoholism**			1.000*			0.897			0.867			0.476
No	**80**	12 (15.0)		**78**	26 (33.3)		**78**	31 (39.7)		**75**	22 (29.3)	
Yes	**23**	3 (13.0)		**23**	8 (34.8)		**24**	10 (41.7)		**23**	5 (21.7)	
**Cancer family history**			0.932			0.901			0.432			0.677
No	**59**	9 (15.3)		**56**	18 (32.1)		**57**	20 (35.1)		**54**	15 (27.8)	
Yes	**41**	6 (14.6)		**42**	14 (33.3)		**42**	18 (42.9)		**41**	13 (31.7)	
**Metastasis dominant site**			**<0.001**			**<0.001**			0.418			0.142
Liver	**42**	2 (4.8)		**42**	7 (16.7)		**43**	15 (34.9)		**41**	10 (24.4)	
Bone	**30**	1 (3.3)		**29**	6 (20.7)		**29**	8 (27.6)		**28**	5 (17.9)	
Lymph node	**27**	9 (33.3)		**27**	16 (59.3)		**27**	12 (44.4)		**27**	11 (40.7)	
**Biopsy site**			**<0.001**			**<0.001**			0.441			0.196
Liver	**38**	1 (2.6)		**37**	4 (10.8)		**38**	14 (36.8)		**35**	9 (25.7)	
Bone	**27**	1 (3.7)		**26**	7 (26.9)		**26**	7 (26.9)		**26**	4 (15.4)	
Lymph node	**31**	11 (35.5)		**31**	17 (54.8)		**30**	13 (43.3)		**30**	11 (36.7)	
**Histological subtype**			0.123			0.098			0.449			0.896
Adenocarcinoma	**66**	5 (7.6)		**64**	15 (23.4)		**64**	23 (35.9)		**62**	19 (30.6)	
Carcinoma	**13**	3 (23.1)		**12**	4 (33.3)		**13**	4 (30.8)		**12**	2 (16.7)	
Squamous cell carcinoma	**8**	3 (37.5)		**9**	5 (55.5)		**9**	6 (66.7)		**9**	3 (33.3)	
Poorly differentiated adenocarcinoma	**8**	1 (12.5)		**8**	2 (25.0)		**8**	3 (37.5)		**7**	2 (28.6)	
Poorly differentiated carcinoma	**15**	2 (13.3)		**15**	8 (53.3)		**15**	5 (33.3)		**15**	4 (26.7)	
**PS (ECOG)**			0.457*			0.457			0.208			0.193
0–1	**47**	6 (12.8)		**45**	14 (31.1)		**45**	19 (42.2)		**43**	15 (34.9)	
≥2	**34**	2 (5.9)		**34**	8 (23.5)		**35**	10 (28.6)		**33**	7 (21.2)	

PS (ECOG), performance status (Eastern Cooperative Oncology Group) (22). *Fisher’s exact test. Significant p-values are depicted in bold.

### Survival Analysis

**Figure 2:**
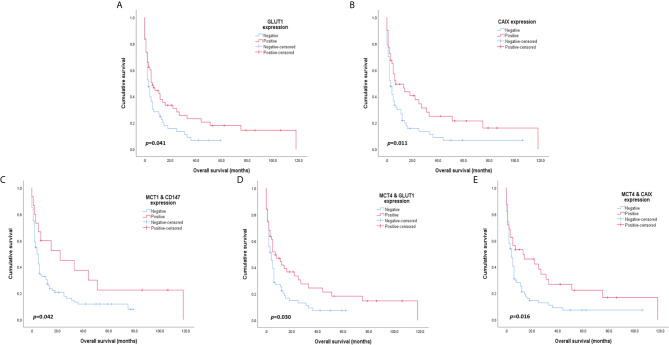
Overall survival curves of cancer of unknown primary origin patients. The red line refers to positive expression of the metabolic proteins presented, and the blue line refers to negative expression. GLUT1 **(A)** and CAIX **(B)** expression was significantly associated with higher patient overall survival. Co-expression of MCT1 and CD147 **(C)** is significantly associated with improved overall survival. Additionally, MCT4 co-expression with GLUT1 **(D)** and CAIX **(E)** was significantly associated with higher overall survival rates in CUP patients.

Overall survival analysis of MCTs, CD147, GLUT1 and CAIX in CUP patients (**Figure 2**) showed that positive GLUT1 and CAIX expression was significantly associated with higher overall survival (*p* = 0.011 and *p* = 0.041, respectively). Regarding the influence of co-expression with the overall survival of CUP patients, MCT1 and CD147, when positively co-expressed, showed better overall survival rates (*p* = 0.042), as well as MCT4/GLUT1 (*p* = 0.030) and MCT4/CAIX (*p* = 0.016) co-expression.

The prognostic values of the proteins and clinicopathological parameters for overall survival were assessed using Cox proportional hazards regression models (**Table 6**). Univariable analysis revealed the prognostic values of the performance status and CAIX expression. Multivariable analysis showed that a performance status ≥2 was an independent prognostic factor for overall survival (HR: 2.352, *p* = 0.002). None of the analyzed metabolism-related proteins presented a prognostic value in the multivariable analysis of CUP patients.

**Table 6:** Prognostic factors for the overall survival of patients with cancer of unknown primary origin.

	Univariable			Multivariable		
	HR	95% CI	*p*	HR	95% CI	*p*
**Age (years)**						
≥59.7	1	-	-			
<59.7	0.819	0.549-1.221	0.327			
**Sex**						
Male	1	-	-			
Female	0.770	0.517-1.148	0.199			
**Smoker**						
No	1	-	-			
Yes	0.921	0.612-1.387	0.694			
**Alcoholism**						
No	1	-	-			
Yes	1.470	0.910-2.374	0.116			
**Cancer family history**						
No	1	-	-			
Yes	0.816	0.530-1.256	0.356			
**PS (ECOG)**						
0 to 1	1	-	-	1	-	-
≥2	2.449	1.482-4.048	**0.001**	2.352	1.368-4.042	**0.002**
**MCT1**						
Negative	1	-	-			
Positive	0.703	0.423-1.169	0.175			
**MCT4**						
Negative	1	-	**-**			
Positive	0.830	0.539-1.277	0.396			
**CD147**						
Negative	1	-	-			
Positive	0.732	0.472-1.136	0.164			
**GLUT1**						
Negative	1	-	-	1	-	-
Positive	0.661	0.434-1.008	0.055	0.930	0.521-1.660	0.807
**CAIX**						
Negative	1	-	-	1	-	-
Positive	0.577	0.368-0.905	**0.017**	0.544	0.296-1.001	0.050

PS (ECOG), performance status (Eastern Cooperative Oncology Group) (22); HR, hazard ratio; CI, confidence interval. Significant p-values are depicted in bold.

## Discussion

Our study demonstrated that the metabolism-related proteins MCT1, MCT4, CD147, GLUT1 and CAIX are expressed in an important portion of the CUP, indicating a hyperglycolytic phenotype. More importantly, we found evidence that this hyperglycolytic behavior is more frequently observed in the CUP located in lymph nodes. Unexpectedly, survival analysis of CUP patients demonstrated that positive expression of GLUT1 and CAIX, their co-expression with MCT4, and MCT1/CD147 co-expression were positively associated with improved survival.

The present study showed expressive plasma membrane expression frequencies of MCT1, MCT4, CD147, GLUT1 and CAIX but not MCT2 in the CUP. During the metabolic adaptation of cancer cells, the dual role of MCT1 and MCT4 in maintaining the glycolytic phenotype by, on the one hand, facilitating lactate efflux and, on the other hand, contributing to the preservation of intracellular pH through co-transport with a proton determines the increased expression of these isoforms in tumor cells (16), as well as that of their chaperone CD147 (17). Furthermore, GLUT1 facilitates glucose uptake, and CAIX, responsible for the reversible conversion of CO_2_ and regulation of intracellular pH, contributes to the acid-mediated cancer cell invasive phenotype, both also contributing to the maintenance of the Warburg effect, particularly in oxygen-depleted (hypoxia) conditions (28, 29). By contrast, MCT2 expression was low, as expected, because MCT2, which is mostly involved in lactate uptake (30), is mainly found in the cytoplasm of tumor cells (31) and is associated with favorable prognostic characteristics in other types of cancers (32, 33). In accordance with the study by Kim and colleagues (20), using a series of 77 CUPs, the frequencies of MCT4 and GLUT1 observed were similar to ours, with MCT4 being present in 71.4% and GLUT1 in 46.8% of samples. However, the authors showed a lower expression frequency of CAIX (11.7%) than our findings. The same study showed an association of GLUT1 expression with the histological subtype of metastasis, with squamous cell carcinomas presenting a higher frequency of expression of this protein. This result suggests a hyperglycolytic phenotype and agrees with our results, showing that MCT1 was more frequently expressed in squamous cell carcinomas, which may be related to aggressiveness, high grade, high proliferative activity and poor prognosis, as observed in other tumor types (16, 33, 34). The study performed by Koo and collaborators (35), in which 69 CUPs were analyzed, also suggests a highly glycolytic profile that determines a worse prognosis in CUP and shows that histological subtypes have different metabolic behaviors. Among other proteins, the authors analyzed the expression of proteins involved in the metabolic reprogramming of cancer cells, such as HIF-1α, GLUT1, phosphorylated mTOR, phosphorylated S6, AMPKα1 and phosphorylated Akt, and identified a higher expression of proteins associated with the hypoxic tumor microenvironment, such as GLUT1 and HIF-1α, in squamous cell carcinomas. Additionally, GLUT1 and HIF-1α expression was associated with a worse prognosis in this histological subtype, with HIF-1α being an independent factor for a worse prognosis (35). Thus, the feasibility of therapeutic approaches targeting metabolic reprogramming of CUP is suggested based on the evidence for metabolic heterogeneity of the different histological subtypes (20, 35). Additionally, MCT1 and CD147 were both more frequently expressed in samples from lymph nodes as well as cancers with lymph nodes as metastasis-dominant sites. A notable characteristic of the lymphatic system is its hypoxic environment, with the absence of red blood cell transport in lymphatic vessels. Generally, lymph vessels are located in remote oxygen-depleted areas, away from oxygen-carrying blood vessels. Tumor cells must adapt to this adverse hypoxic environment to sustain lymphangiogenesis and spread to lymph nodes, particularly with the upregulation of VEGF-C mediated by HIF-1α activation (36).

In the search for a metabolic profile with clinical significance in CUP and a better understanding of this tumor entity, we evaluated co-expression between the proteins, and a significant association was found for MCT1/CD147, MCT4/CD147, MCT4/GLUT1 and MCT4/CAIX co-expression. This finding suggests a hyperglycolytic and acid-resistant phenotype in a subset of CUPs, sustaining the Warburg effect for cell proliferation and cancer cell survival (11, 12, 37–40). MCT1/CD147 and MCT4/CD147 co-expression was significantly associated with the lymph node biopsy site and the lymph node as the metastasis dominant site. These results were also found individually for MCT1 and CD147, supporting the synergism characteristic among MCT1, MCT4 and CD147 in the context of solid tumors related to microenvironment acidification and an increased metastatic potential (34, 41). The co-expression of MCTs with CD147 was previously described in colorectal tumor samples, including lymph node and hepatic metastasis, compared with adjacent normal tissues (42). The study highlights that MCT1/CD147 co-expression was observed in colorectal primary tumors, while MCT4/CD147 co-expression was observed in colorectal primary tumor, lymph node and hepatic metastasis samples (42). Thus, the co-expression findings of the present study are consistent with the biological role of these proteins because CD147 is a chaperone of MCT1 and MCT4 and is often co-expressed in tumor cells (42–44).

Unexpectedly, our results demonstrated that CUP patients with positive GLUT1 and CAIX expression presented significantly higher overall survival, and the same was observed for the co-expression of these proteins with MCT4 and MCT1/CD147 co-expression. Currently, most studies in the literature have shown that hyperglycolytic tumors present a poorer prognosis and decreased survival rates (38, 39, 45, 46). Conversely, our results indicate a different behavior of this metabolic phenotype in CUP. First, we must consider that other isoforms of glucose transporters and carbonic anhydrases, such as GLUT3 and CAXII, may be present and active in these tumors, similar to other neoplasms (47–53). However, because of sample limitations, we did not characterize the expression of these proteins in our samples, while studies considering the expression of GLUT3 and CAXII in CUP are lacking. Additionally, positive GLUT1 and CAIX expression does not influence the prognostic outcomes related to the overall and disease-free survival of cervical cancer patients (54). Cleven and co-workers (55) showed that positive GLUT1 expression was related to better survival in patients with colorectal tumors. Additionally, CAIX stromal and tumor expression was not related to clinical survival, with CAIX tumor expression showing decreased expression with advancing TNM and that earlier TNM stage has a more pronounced CAIX expression and better survival than later TNM stages. Furthermore, the authors demonstrated that stromal CAIX expression improved the survival rates of colorectal cancer patients, suggesting that tumor hypoxia may influence tumor-associated stromal cells that ultimately contributes to patient prognosis. Although the samples used in the present study were cancer treatment naïve, some studies demonstrated that systemic therapy alters the metabolic phenotype of cancer cells and affects the patient outcomes (25, 56, 57). Goos and co-workers (56) showed that high GLUT1 expression levels in colorectal cancer liver metastasis were associated with improved survival of patients previously treated with systemic therapy. Additionally, a study performed by our group with locally advanced breast tumor patients submitted to neoadjuvant chemotherapy showed that CAIX-positive expression was associated with higher disease-free survival and disease-specific survival, and a pathological complete response after treatment (25), suggesting that neoadjuvant therapy affects the metabolic phenotype of aggressive glycolytic tumors, favoring the clinical outcomes of breast cancer patients. Regarding the association of MCT1/CD147, GLUT1/MCT4 and CAIX/MCT4 co-expression with higher overall survival of CUP patients, to our best knowledge, this is the first report of MCT association with a good clinical outcome; however, it follows the rationale behind GLUT1 and CAIX findings, with glycolytic CUPs showing a better prognosis.

The main limitation of this study is related to the sample heterogeneity due to material from different biopsy sites as a result of metastases with different locations, influencing the analysis, particularly survival analysis. This situation leads to case loss, both during the selection of patients and immunohistochemical procedure due to the reduced tissue size and loss of representative tumor tissue perceived during immunohistochemical evaluation. The aforementioned impacted the total sample size and subgroup size, limiting the analysis considering the variables associated with the heterogeneous profile. Notably, the impossibility of comparing the metabolic profile of CUP samples with the normal tissue of origin is a limitation because the absence of a primary tumor defines this neoplastic entity. However, given the scarcity of previous studies characterizing cellular metabolism in CUP, this study represents an important gain for the literature.

In conclusion, although CUPs are biologically complex, our results suggest that a portion of these tumors present an increased expression of proteins related to glycolytic metabolism, with hyperglycolytic CUPs being associated with higher overall survival. New studies evaluating the metabolic profile of CUPs, as well as the characterization of other metabolism-related proteins and their activity, could contribute to a better understanding of this enigmatic entity of tumors, enhancing the clinical diagnosis, management and determination of new prognostic markers and therapeutic strategies.

## Data Availability Statement

The raw data supporting the conclusions of this article will be made available by the authors, without undue reservation.

## Ethics Statement

The studies involving human participants were reviewed and approved by the Barretos Cancer Hospital Ethics Committee. Written informed consent for participation was not required for this study in accordance with the national legislation and the institutional requirements.

## Author Contributions

MB, IF, PB and LP performed immunohistochemical reactions and wrote the manuscript. JJ, IF, PB and LP retrieved the clinicopathological data of patients from medical records. RC performed the statistical analysis. ES and AL-F analyzed the histological sections and performed the immunohistochemical evaluations. FC aided in the study design and discussion of the results. CP was responsible for the study design, contributed to the discussion of the results, organization and review of the manuscript. All authors contributed to the article and approved the submitted version.

## Funding

This study was supported by FAPESP, Fundação de Amparo à Pesquisa do Estado de São Paulo (2016/08674-9; 2017/09226-2) and CNPq, Conselho Nacional de Desenvolvimento Científico e Tecnológico (306195/2016-0, 309998/2019-0).

## Conflict of Interest

The authors declare that the research was conducted in the absence of any commercial or financial relationships that could be construed as a potential conflict of interest.
